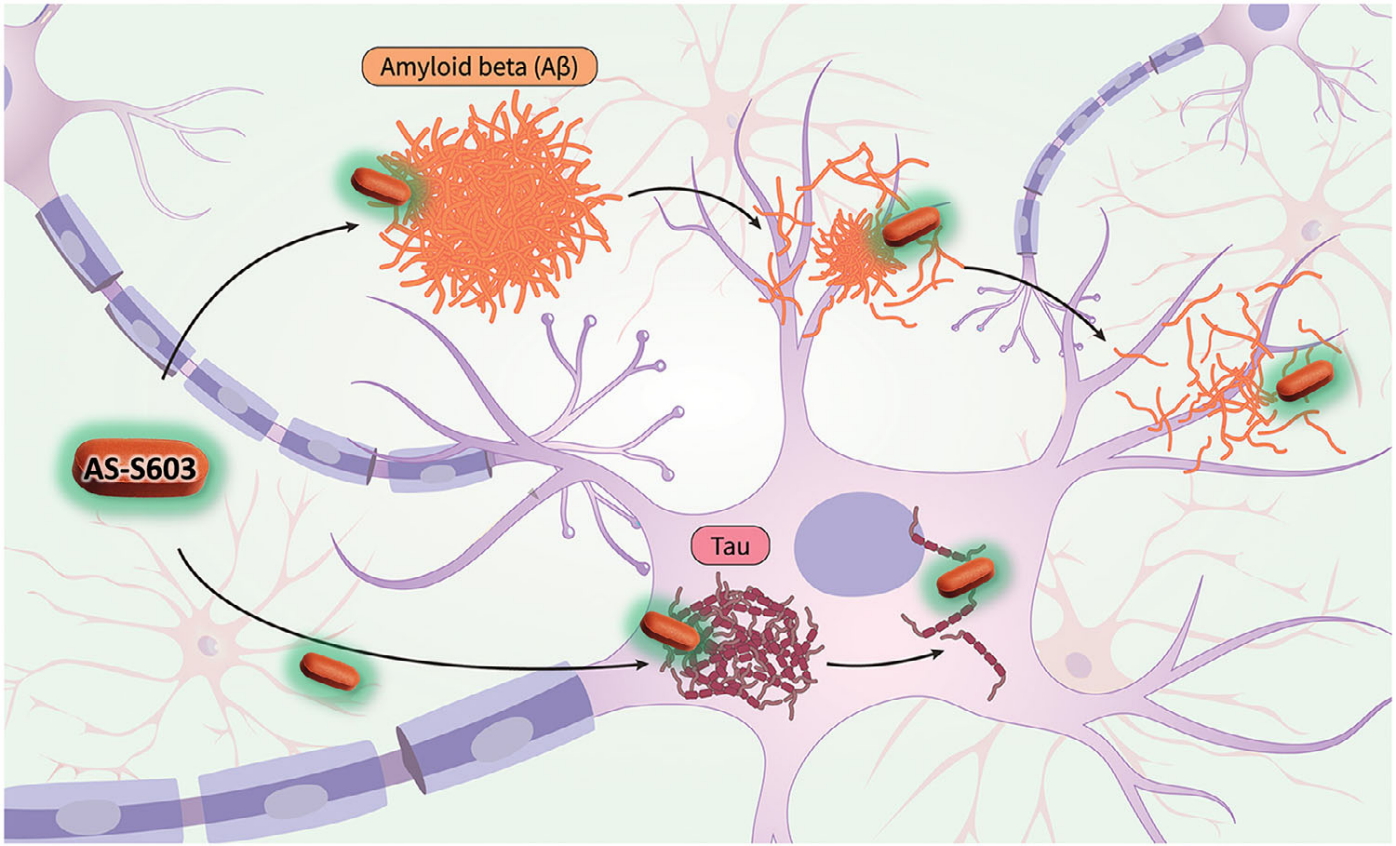# Chemical‐Driven Clearance of Aβ and tau Aggregates by Orally Available Small Molecule Drug Candidate AS‐S603

**DOI:** 10.1002/alz70859_100122

**Published:** 2025-12-25

**Authors:** YoungSoo Kim, Seung Hoon Han, Hyemi Lee, Yoon Sun Chun, Seong Muk Kim, Hye‐Ju Kim, Seong Jin Park

**Affiliations:** ^1^ Amyloid Solution Inc., Seongnam, Gyeonggi‐do Korea, Republic of (South); ^2^ College of Pharmacy, Yonsei University, Incheon, Incheon Korea, Republic of (South)

## Abstract

**Background:**

Alzheimer’s disease (AD) is characterized by the accumulation of amyloid‐β (Aβ) plaques and tau tangles, key therapeutic targets. While immunotherapies have been developed to clear Aβ or tau aggregates individually, no strategy has simultaneously targeted both. AS‐S603, a novel small molecule in clinical trial phase 1 (NCT06786676), is designed to chemically clear Aβ and tau aggregates, including plaques, tangles, and oligomers, by dissociating them into monomers and preventing further aggregation.

**Method:**

AS‐S603 was discovered through an unbiased high‐throughput screening, a proprietary assay system designed to identify molecules that dissociate misfolded proteins from over 50,000 compounds targeting Aβ and tau aggregates. Hits were optimized through iterative derivatization to the final candidate. Comparative studies with Lecanemab, ALZ‐801, and TRx0237 in aged 5xFAD and PS19 mice highlighted its efficacy, including behavioral improvements in Morris Water Maze tests and amyloid clearance imaging with amyloid‐PET using 18F‐fluorobetaben. Novel tools were developed to investigate its mode of action (MoA), revealing how AS‐S603 dissociates oligomers, plaques, and tangles with specificity to Aβ and tau. Non‐clinical studies confirmed its tolerability and clinical readiness. For clinical trials, AS‐S603 was developed in a once‐daily tablet.

**Result:**

AS‐S603 dissociates Aβ and tau aggregates, converting both soluble oligomers and insoluble plaques and tangles into monomers. It targets extracellular and intracellular aggregates, broadening its therapeutic scope. In preclinical studies, AS‐S603 showed significantly improved cognitive benefits compared to Lecanemab at both 1 mpk and 10 mpk. Amyloid‐PET scans revealed a more substantial reduction in amyloid burden for AS‐S603. Biophysical, biochemical, and molecular docking simulation studies revealed its MoA, showing that AS‐S603 binds to the hydrophobic pocket of Aβ and tau, disrupting hydrogen bonding and inducing dissociation. Binding assays showed no off‐target effects. Blood‐brain barrier penetration of AS‐S603 is over 200%, demonstrating an advantage over antibodies. Safety evaluations confirmed a favorable tolerability profile. The Phase 1 clinical trial is currently underway.

**Conclusion:**

AS‐S603 is a first‐in‐class oral treatment for AD, uniquely targeting both Aβ and tau aggregates. It provides significant cognitive benefits in preclinical studies, suggesting its potential to alter disease progression. Clinical studies will validate its safety and efficacy, positioning AS‐S603 as a transformative treatment for AD.